# An ileo-colic intussusception reaching down to the descending colon - A case report

**DOI:** 10.1016/j.ijscr.2022.107009

**Published:** 2022-04-02

**Authors:** Hugo Teixeira, Fabian Hauswirth, Nina Römer, Markus K. Muller, Matthias Baechtold

**Affiliations:** Department of Surgery, Cantonal Hospital Frauenfeld, Pfaffenholzstrasse 4, 8501 Frauenfeld, Switzerland

**Keywords:** Intussusception, Bowel obstruction, colon resection, Case report

## Abstract

**Introduction and importance:**

Intussusception in healthy adults is rare and often associated with oncologic diseases. This case report presents a case of an ileo-colic intussusception reaching down to the descending colon in a healthy adult that required ileo-colic resection.

**Case presentation:**

We present a case of a 78-year-old male patient with acute onset unspecific abdominal pain. The medical history was unremarkable. Preoperative radiologic assessments showed an invagination of the small intestine into the colon without any signs of polyps or tumours. An emergency laparotomy with resection of the affected intestine was performed. The pathologist described a 49 cm length of intussuscepted colon and an additional 7 cm intussusception of the terminal ileum. A circular area with multiple polyps extending over 8 cm in the colon could be identified. The microscopic findings showed a low-grade dysplasia within this area. Following surgery, the patient was discharged to rehabilitation after a ten-day hospitalization.

**Clinical discussion:**

Intussusception in adults is rare and the clinical presentation includes unspecific symptoms making the diagnosis challenging. In 90% of the cases, a pathologic lesion is found (two-thirds are neoplasms). An intussusception involving the colon should be treated surgically without prior reduction due to the high incidence of a neoplasm and the risk for perforation and tumour dissemination.

**Conclusion:**

In the literature, neoplastic disease represents the major cause for intussusception in adults. This report presents a rare case of an ileo-colic intussusception reaching down to the descending colon treated successfully with a subtotal colectomy.

## Highlights

• Intussusception is a differential diagnosis in adults with abdominal pain.
• In up to 90% of the cases, an underlying disease or condition can be identified.
• Increasing incidental diagnosis due to better quality and availability of CT scan.
• If the colon is involved, the best treatment is surgical without prior reduction.

## 1 Introduction

Intussusception is a rare finding in adults and is responsible for less than 5% of mechanical bowel obstruction [1], [2]. Most of the available literature describes intussusception as the result of an underlying disease or condition that is identifiable in up to 90% of the cases. The most common underlying cause for intussusception is described to be a neoplastic disorder, which accounts for two-thirds of cases [3].

To our knowledge, no other case report presented a case of such an ileo-colic intussusception in an otherwise healthy adult.

The patient was informed that data concerning the case would be submitted for publication and provided consent.

The work has been reported in line with the SCARE 2020 criteria [4].

## 2 Presentation of case

A 78-year-old male patient walked into our emergency department with a 3-day history of diffuse abdominal pain accompanied by nausea, vomiting, and diarrhoea. He denied having had fever or any history of gastrointestinal bleeding. Since the onset of his complaints, the patient had loss of appetite. His weight was stable in the last year. Otherwise, the medical history, including genetic information, was unremarkable and the patient was under no regular medication. The family history, as well as the psycho-social history, were unremarkable. The patient never had a colonoscopy nor had he ever undergone abdominal surgery before. No further relevant interventions were reported.

**Fig. 1:**
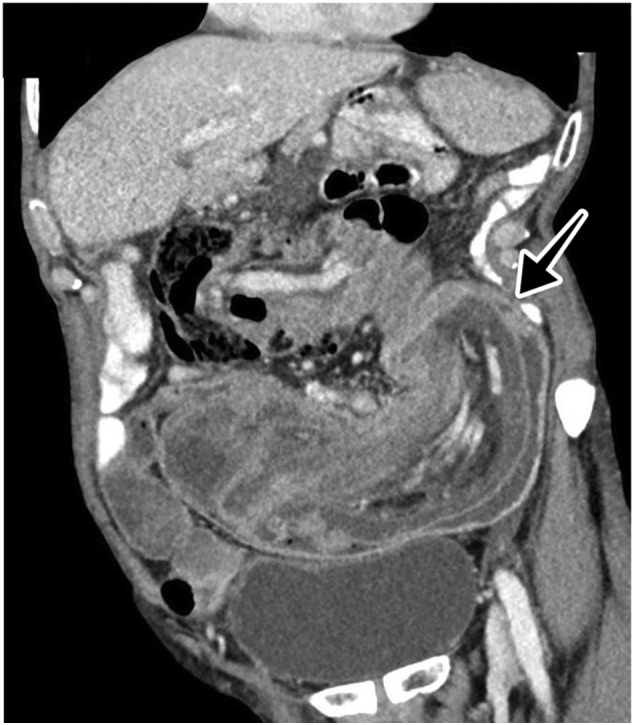
CT scan of the abdomen. Coronal plane showing an ileo-colic intussusception with classic ‘sausage-shaped mass’ (arrow).

**Fig. 2:**
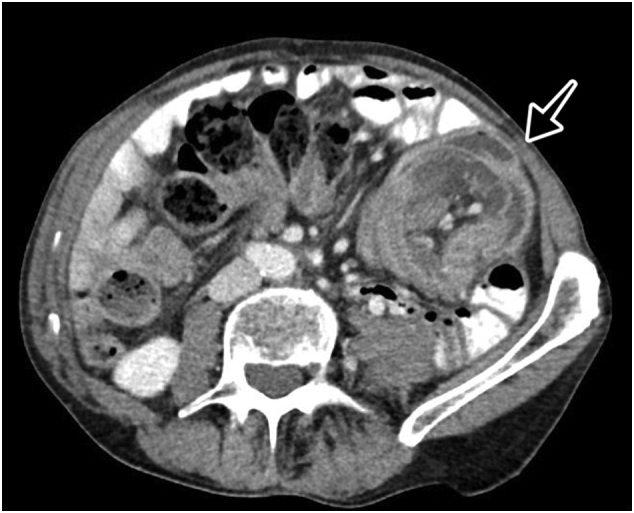
CT scan of the abdomen. Axial plane showing an ileo-colic intussusception with the classic ‘bulls-eye’ appearance (arrow).

Examination of the abdomen showed a periumbilical palpable mass extending towards the right lower quadrant. The mass was not tender, and there were no sign of peritonitis. Laboratory testing provided slightly elevated inflammatory markers and negative tumour markers. The following computed tomography (CT) showed a pronounced invagination of the small intestine and the colon (Fig. 1, Fig. 2). There were no signs of inflammatory processes or malignancies. These findings were indicative of an ileo-colic intussusception.

The patient was prepared for emergency explorative laparotomy, which was performed by a senior consultant. Intraoperatively, we found an ileo-colic intussusception that could be palpated down to the descending colon. There were no signs of intestinal ischemia, peritoneal carcinosis, or suspicious mesenterial lymph nodes. The colon wall was fragile and at risk of tearing during the manipulation. We decided to perform a colectomy of the affected colon and the terminal ileum. This resulted in a subtotal colectomy with en-bloc resection according to oncological principles. The bowel continuity was restored with a side-to-side anastomosis of the terminal Ileum and the descending colon, and the abdominal wall was closed in the usual manner. Postoperatively, the patient underwent gradual progression from liquids to solids and was discharged after ten days of hospitalization.

**Fig. 3:**
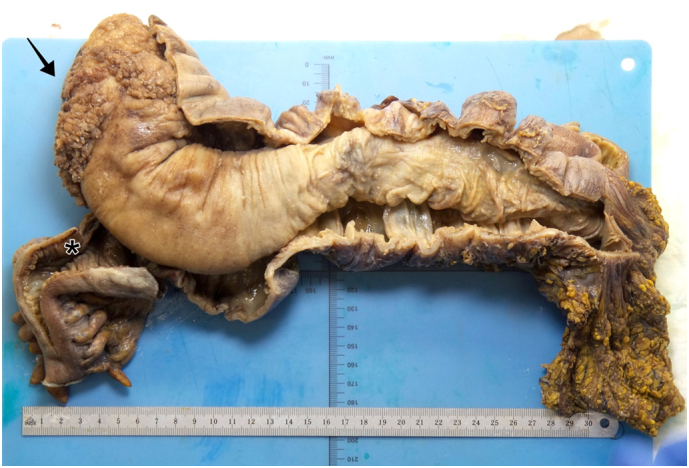
Surgical specimen during pathological examination and after incision of the colon wall showing the intussusception reaching down to the descending colon (asterisk). The circular area with multiple polyps (arrow) acted as the leading point.

In the histopathological examination, the pathologist macroscopically described 49 cm of intussuscepted colon with a diameter of 4 cm (Fig. 3). The emerging part of the intussuscepted ileum was 7 cm in length. The terminal ileum was extensively intussuscepted. In the microscopic examination, the colon mucosa presented with an 8 cm long, circular area of polypoid changes that could be defined as the leading point for the intussusception. Inside this area, round nuclei with vesicular chromatin were found (all signs of a potential low-grade dysplasia without clues for malignancies). There was no lymphatic metastasis in the 21 resected nodes, and the resection margins were negative. Prior to surgery, no colonoscopy was performed due to increased risk for wall perforation. However, we recommended a follow-up colonoscopy that was organized by the general physician. The general physician followed up with the patient after discharge and has not reported any relevant adverse events.

## 3 Discussion

This case presents a rare example of an ileo-colic intussusception reaching down to the descending colon in an otherwise healthy patient who presented with unspecific abdominal pain. Based on the palpable intraabdominal mass and the patient's age, we suspected a growing tumour. Some differential diagnoses were also taken into consideration. An acute appendicitis, especially in elder patients, might present with similar symptoms, and the absence of fever does not rule out an appendicitis [5], [6]. Further, small bowel obstruction would most likely originate due to adhesions from previous surgeries, followed by hernias [7], [8], [9]. Our patient did not have a history of previous surgeries and the abdominal wall appeared intact. When it comes to large bowel obstruction, diverticulitis or colonic volvulus are less common causes, but the work-up needs to take them into consideration. During our clinical work up, the tumour markers were negative and the CT scan showed an ileo-colic intussusception.

The anatomical presentation of an intussusception varies widely: gastro-duodenal, jejuno-jejunal, ileo-ileal, ileo-colic, and colo-colic. In 90% of cases, a pathologic lesion is found—most of them intraluminal [10]. Two-thirds of the cases are due to a neoplasm (up to 50% of these cases are malignant). The remaining cases might be caused by infections, adhesions, or other non-neoplastic causes. Intussusceptions are more common in the small bowel compared to the colon, and the most common malignant cause of intussusception is a primary colonic adenocarcinoma [1], [10], [11].

The paediatric patients with intussusception can present with the triad of acute onset colicky pain, palpable abdominal mass, and—with disease progression—bloody stool. This triad, however, is described in less than 15% of cases [3]. In the adult patient, symptoms are even more unspecific and include subacute or chronic symptoms, such as abdominal pain, nausea, vomiting, bleeding, or constipation [1], [3], [10]. These unspecific presentations increase the difficulty of diagnosing intussusception in adult patients. Given the vast potential differentials, imaging techniques might support the clinician to rule out some diagnoses.

Plain radiograph may reveal signs of obstruction and its location, but they lack sensitivity and specificity for the diagnosis of intussusception [12]. Sonography has been described with a diagnostic accuracy of just under 50% [13]. The most sensitive diagnostic tool is the CT of the abdomen, which might show the pathognomonic target and sausage-shaped signs [14], [15]. Further, the precise location and the cause of the intussusception might be visible following the CT. It has been reported that a CT scan is positive for 93% of adults with intussusception [16]. With the increased quality and availability of CT in the diagnosis of abdominal pathology, the number of incidentally diagnosed intussusceptions in patients being scanned for another reason, as well as those without an identifiable lead point, has also increased [14], [17].

Regarding the best treatment, the literature remains controversial due to the different aetiologies in children and adults. In the literature, adult intussusception has been primarily treated surgically with resection of the affected bowel segment due to the prevalence of a malignancy as a leading point [3]. Based on the literature, not only the presence of a mass in the imaging as a suspected leading point, but also the presence of bowel obstruction, is significantly associated with a malignant neoplasm and should be treated surgically [14]. Nonetheless, there is still controversy, since the use of modern radiological exams can help distinguish between an intussusception with and without a leading point, thus preventing unnecessary surgery [18]. Furthermore, evidence supports that asymptomatic incidental intussusceptions, without a leading point or signs of obstruction, are likely due to transient abnormalities in the peristalsis and are therefore likely to be successfully treated nonoperatively [19]. Onkendi et al. reported in their retrospective study that in 19% of the cases, the patients were asymptomatic at the time of presentation, and the intussusception was incidentally found in the imaging. These patients were successfully treated nonoperatively, and the intussusception reduced spontaneously and could not be seen in further imaging [14].

If a benign lesion has been diagnosed and the bowel is viable or the resection will lead to short gut syndrome, one should consider a preoperative reduction. A colonoscopy can also be pursued to confirm the pathology or malignancy [3], [10], [20]. Even so, there seems to be a consensus in the literature that a intussusception involving the colon should be treated surgically without prior manipulation or reduction, especially in patients older than 60 years old, due to the higher risk of intraluminal seeding and tumour dissemination, perforation and seeding of tumour cells in the abdominal cavity, venous dissemination of tumour cells, and increased risk of anastomotic complication [1], [10], [20]. The surgical resection can be performed laparoscopically or open, depending on the surgeon's experience. Irrespective of the approach, the resection must follow strict oncological principles. A primary anastomosis can be performed if the tissue seems viable [10], [20].

In our case, the patient was diagnosed with an ileo-colic intussusception, and we performed an emergency laparotomy and resected the affected bowel segment, following the oncological principles. In the histopathologic findings, there was evidence of a circular area extending over 8 cm with multiple polyps as a leading point for the invagination with microscopical evidence of a small area with low-grade dysplasia without lymphatic metastasis and negative resection margins. There were no postoperative complications during the stay at the hospital or after discharge.

As a learning point, we believe that the description of this case, alongside review of the literature, may not only raise awareness to intussusception as a differential diagnosis on a setting of abdominal pain but also help keep in mind that even in such a case the basic principles of surgery are applied.

## 4 Conclusion

Elderly patients presenting with abdominal mass, sub-ileus symptoms, and loss of appetite would raise the suspicion of a malignant disease, especially in patients who did not undergo prior routine screening for colon cancer. However, the rare diagnosis of intussusception represents a valid differential and should be considered in patients with these symptoms.

## Provenance and peer review

Not commissioned, externally peer-reviewed.

## Sources of funding

No external funding was received for this study.

## Ethical approval

This case report is exempt from ethical approval in our institution.

## Consent

Written informed consent was obtained from the patient for publication of this case report and accompanying images. A copy of the written consent is available for review by the Editor-in-Chief of this journal upon request.

## Author contribution

Hugo Teixeira treated the patient and wrote the original article. Fabian Hauswirth and Nina Römer treated the patient and critically reviewed the article; Markus K. Muller critically reviewed the article; Matthias Baechtold treated the patient, supervised and critically reviewed the article.

## Research registration

Not applicable.

## Guarantor

Hugo Teixeira.

## Declaration of competing interest

None of the authors declared any conflicts of interest.

## References

[1] Azar T., Berger D.L. (1997). Adult intussusception. Ann. Surg..

[2] Marinis A., Yiallourou A., Samanides L., Dafnios N., Anastasopoulos G., Vassiliou I. (2009). Intussusception of the bowel in adults: a review. World J. Gastroenterol..

[3] Marsicovetere P., Ivatury S.J., White B., Holubar S.D. (2017). Intestinal intussusception: etiology, diagnosis, and treatment. Clin. Colon Rectal Surg..

[4] Agha R.A., Franchi T., Sohrabi C., Mathew G., for the SCARE Group (2020). The SCARE 2020 guideline: updating consensus Surgical CAse REport (SCARE) guidelines. Int J Surg.

[5] Kraemer M., Franke C., Ohmann C., Yang Q., Acute Abdominal Pain Study Group (2000). Acute appendicitis in late adulthood: incidence, presentation, and outcome. Results of a prospective multicenter acute abdominal pain study and a review of the literature. Langenbeck's Arch. Surg..

[6] Elangovan S. (1996). Clinical and laboratory findings in acute appendicitis in the elderly. J. Am. Board Fam. Pract..

[7] Sanson T.G., O'Keefe K.P. (1996). Evaluation of abdominal pain in the elderly. Emerg. Med. Clin. North Am..

[8] Vogt D.P. (1990). The acute abdomen in the geriatric patient. Cleve. Clin. J. Med..

[9] Hendrickson M., Naparst T.R. (2003). Abdominal surgical emergencies in the elderly. Emerg. Med. Clin. North Am..

[10] Eisen L.K., Cunningham J.D., Aufses A.H. (1999). Intussusception in adults: institutional review. J. Am. Coll. Surg..

[11] Wang L.T., Wu C.C., Yu J.C., Hsiao C.W., Hsu C.C., Jao S.W. (2007). Clinical entity and treatment strategies for adult intussusceptions: 20 years' experience. Dis. Colon Rectum.

[12] Mrak K. (2014). Uncommon conditions in surgical oncology: acute abdomen caused by ileocolic intussusception. J. Gastrointest. Oncol..

[13] Honjo H., Mike M., Kusanagi H., Kano N. (2015). Adult intussusception: a retrospective review. World J. Surg..

[14] Onkendi E.O., Grotz T.E., Murray J.A., Donohue J.H. (2011). Adult intussusception in the last 25 years of modern imaging: is surgery still indicated?. J. Gastrointest. Surg..

[15] Gayer G., Hertz M., Zissin R. (2003). CT findings of intussusception in adults. Semin Ultrasound CT MR.

[16] Lindor R.A., Bellolio M.F., Sadosty A.T., Earnest F., Cabrera D. (2012). Adult intussusception: presentation, management, and outcomes of 148 patients. J. Emerg. Med..

[17] Valentini V., Buquicchio G.L., Galluzzo M., Ianniello S., Di Grezia G., Ambrosio R. (2016). Intussusception in adults: the role of MDCT in the identification of the site and cause of obstruction. Gastroenterol. Res. Pract..

[18] Kim Y.H., Blake M.A., Harisinghani M.G., Archer-Arroyo K., Hahn P.F., Pitman M.B. (2006). Adult intestinal intussusception: CT appearances and identification of a causative lead point. Radiographics.

[19] Aydin N., Roth A., Misra S. (2016). Surgical versus conservative management of adult intussusception: case series and review. Int. J. Surg. Case Rep..

[20] Takeuchi K., Tsuzuki Y., Ando T., Sekihara M., Hara T., Kori T., Kuwano H. (2003). The diagnosis and treatment of adult intussusception. J. Clin. Gastroenterol..

